# The influence of a MOBile-based video Instruction for Low back pain (MOBIL) on initial care decisions made by primary care providers: a randomized controlled trial

**DOI:** 10.1186/s12875-021-01549-y

**Published:** 2021-10-09

**Authors:** Daniel I. Rhon, Rachel J. Mayhew, Tina A. Greenlee, Julie M. Fritz

**Affiliations:** 1 Department of Rehabilitation Medicine, Brooke Army Medical Center, JBSA Fort Sam Houston, San Antonio, TX USA; 2 Uniformed Services University of the Health Sciences , Bethesda, MD USA; 3 University of Utah, Salt Lake City, UT USA

## Abstract

**Background:**

Adherence to guidelines for back pain continues to be a challenge, prompting strategies focused on improving education around biopsychosocial frameworks.

**Objective:**

Assess the influence of an interactive educational mobile app for patients on initial care decisions made for low back pain by the primary care provider. The secondary aim was to compare changes in self-reported pain and function between groups.

**Methods:**

This was a randomized controlled trial involving patients consulting for an initial episode of low back pain. The intervention was a mobile video-based education session (Truth About Low Back Pain) compared to usual care. The app focused on addressing maladaptive beliefs typically associated with higher risk of receiving low-value care options. The primary outcome was initial medical utilization decisions made by primary care practitioners (x-rays, MRIs, opioid prescriptions, injections, procedures) and secondary outcomes included PROMIS pain interference and physical function subscales at 1 and 6 months, and total medical costs.

**Results:**

Of 208 participants (71.2% male; mean age 35.4 years), rates of opioid prescriptions, advanced imaging, analgesic patches, spine injections, and physical therapy use were lower in the education group, but the differences were not significant. Total back-related medical costs for 1 year (mean diff = $132; *P* = 0.63) and none of the 6-month PROMIS subscales were significantly different between groups. Results were no different in opioid-naïve subjects. Instead, prior opioid use and high-risk of poor prognosis on the STarT Back Screening Tool predicted 1-year back pain-related costs and healthcare utilization, regardless of intervention.

**Conclusion:**

Factors that influence medical treatment decisions and guideline-concordant care are complex. This particular patient education approach directed at patients did not appear to influence healthcare decisions made by primary care providers. Future studies should focus on high-risk populations and/or the impact of including the medical provider as an active part of the educational process.

**Trial Registration:**

clinicaltrials.gov NCT02777983.

## Background

While clinical practice guidelines exist to help drive high-value care and improve outcomes for patients with low back pain (LBP), there continue to be implementation challenges. Low-value care provides no net health benefit in specific clinical scenarios [1]. For LBP expensive tests (e.g. MRI or CT), procedures (e.g. injections, nerve ablations), and therapies (e.g. opioids) deliver limited benefits in terms of reduced pain and increased function [2], especially when part of the initial treatment strategy. The popularity and abundant use of these low-value care components is one reason that low back and neck pain has the highest amount of health care spending out of 154 medical conditions in the US [3]. Strategies to reduce low-value care for LBP continue to be of utmost importance [4].

Patient expectations have been identified as a potential contributor to low-value care, and many of these expectations are based on a biomedical perspective [5–7]. The biomedical approach focuses on biological mechanisms as the primary source of symptoms (e.g. a bulging disc, fracture, malalignment of the spine, etc.), which often places patients in a position of perceived frailty and vulnerability [8]. This has the consequence of ignoring or minimizing psychological, social, and environmental influences [9]. The latter are considered more predictive of long-term disability and chronicity [10]. Most procedures, diagnostic tests, and opioids focus on finding or treating a biomedical problem, but are not considered high-value care when used as initial treatment options. In fact, they can often place patients at higher risk for poor recovery. For example, an MRI is likely to show some variance of abnormality even though imaging findings of any kind are unlikely to change the treatment plan or prognosis [11]. A specific pathoanatomical diagnosis cannot be reliably identified in most cases [11] and is not needed to deliver effective care. Incidental findings may even lead to unnecessary and high-risk interventions [12]. However, many of these high-risk treatment options are associated with higher patient satisfaction [13, 14], further complicating treatment decisions for clinicians. Imaging can reduce anxiety [15] as patients expect a clear and specific diagnosis [16, 17], that some feel is not possible without an MRI [6]. However, because of over-reliance on the biomedical paradigm, patients also interpret a decision to forego medical treatment they think important (imaging, procedures, medication) as being associated with low-value care [18] or even as care that physicians for a variety of reasons are trying to ration [19]. Doctors report ordering unnecessary tests because of pressure from patients [20], and are more likely to prescribe opioids when they have less time to address psychological, environmental, and social variables that are commonly related to poor prognosis with back pain [21].

Patient expectations are often driven by misinformation, often placing clinicians in a conflicted position of choosing to satisfy patients versus following guideline recommendations. In a study of 130 patients, 89% reported learning many of their misconceptions about back pain from previous health care providers, which provides some insight as to why these beliefs are so difficult to unravel [22]. Mass media campaigns that leverage psychosocial paradigms to improve the public’s health literacy related to misconceptions about the biomedical causes of back pain have been called for [23], but the magnitude of their effect is questionable leaving room for improvement [24, 25]. Focusing on the individual patient at the point of care with a more engaging format, right when the problem is most pressing, could provide another influential opportunity for education.

Equipping patients with appropriate and engaging information that shifts the focus from the biomedical perspective to one that addresses psychosocial risk factors has the potential to improve high-value care, and ultimately long-term outcomes. The purpose of this study was to compare initial treatment choices for LBP made by primary care clinicians based on whether the patient was primed with an educational session focused on addressing biomedically-focused misconceptions about the diagnosis, treatment, and prognosis for LBP immediately before seeing the primary care provider. Would a patient receiving this information help influence high-value care decisions for LBP made by the primary care provider? The secondary aim was to compare changes in self-reported pain and function between the two groups from baseline to 6 months, and total back pain-related healthcare costs during the full year after the initial diagnosis.

## Methods

### Design and trial oversight

This was a parallel group randomized controlled trial with a 1:1 allocation to treatment. Ethics approval was provided by the Institutional Review Board at Army Regional Health Command Central, the trial was registered a priori (clinicaltrials.gov NCT02777983; 05/19/2016), and the CONSORT checklist was used to guide reporting [26].

### Setting and participants

Participants were individuals between the ages of 18 and 50 consulting for low back pain in a hospital-based primary care clinic in San Antonio, TX. This age range was chosen as it best aligns with the age range of military personnel. Individuals seeking care in this setting are TRICARE beneficiaries; covered by the health system under the US Defense Health Agency. Participants were excluded if they had prior spine surgery, were currently or recently pregnant within the last 6 months, had any non-musculoskeletal cause for symptoms (severe neurological deficit, fracture, cancer, infection, or other systemic disease), had already sought care for their back pain in the last 3 months, or did not read or write in English. Because the intervention occurred in the Military Health System, and to try and make the findings relevant to a service member population, individuals under the age of 18 or over the age of 50 were also excluded.

### Randomization

A randomization sequence was developed by an individual at our partner university that was not participating in the trial in permuted blocks of four. The treatment allocation was written on a 3 × 5 index card, folded in half, and placed in a sealed opaque envelope. This stack of sequentially numbered envelopes was then given to the research team and an envelope was opened by a research coordinator after a participant had enrolled in the study and completed all baseline assessments.

### Interventions

The intervention is described in detail according to the Template for Intervention Description and Replication (TIDieR) checklist (Table 1) [27]. Participants were randomized to receive either a guided video-based education session that focused on shifting pain-related attitudes and beliefs from an unhealthy biomedical focus to a more holistic biopsychosocial focus (video from Truth About Low Back Pain) [28] or usual care. The video education also provided a unique and more engaging format to deliver this content (comprehension questions asked at the end, key points reviewed, etc.) compared to traditional books or print media [29]. For individuals randomized to the usual care group, nothing was done differently to influence education or care above and beyond what they would normally receive from the medical staff during their appointment. Participants in the education group were taken into a private room by a credentialed clinician (not their primary care provider), allowed to fully watch the video in the app, answer the related quiz questions, review sections for questions answered incorrectly, and ask additional questions while the clinician reinforced the key messages:

1. Most low back pain resolves in several weeks’ time regardless of pain severity.
2. MRIs and x-rays are not much help in most cases.
3. Over the counter medications should be used sparingly and narcotics should be avoided completely.
4. The two most important things you can do for your back pain are to 1) Stay Active, and 2) Think Positive.

**Table 1:** Description of the MOBile-based Instruction for Low back pain (MOBIL) intervention according to the TIDier checklist [26]

Item Name/Number	Item Description
**Item 1: BRIEF NAME** MOBIL	
**Item 2: WHY** Interactive tools focused on educating before the clinical encounter have the potential to help providers adhere to clinical practice guidelines (CPGs) by shifting focus and agency from provider to patient. Adequate information that shifts the focus from the traditional biomedical focus to a more psychosocial aspect of back pain and recovery could put less pressure on a provider to deliver low-value care. Timely education about appropriate self-management strategies provided before the patient sees their provider could equip patients with pertinent information that a short clinic visit often has no time to address.	
**Item 3: WHAT, MATERIALS** A. MOBile based Instruction for Low back pain (all information and content found in mobile app) [27] delivered on a clinic iPad tablet computer.	
Section 1: Home Section 2: Video Section 3: Quiz Section 4: Bottom Line Section 5: More Info About MOBIL Handout B. Usual Care	“The content of this tool is based on the best available medical research on low back pain. Its purpose is to produce useful knowledge that YOU can use today to maximize your recovery.” Content includes these main topics: 1) very few cases of LBP are attributable to serious pathology 2) reassurance that a diagnostic label is not necessary for effective treatment 3) reassurance that very few individuals benefit from imaging procedures 4) emphasis on the beneficial effects of remaining active even with some persistent pain 5) the generally favorable prognosis for an episode of LBP when activity is maintained Contains 6 multiple choice questions intended to reinforce the video’s content each with individual links to review short video segments to provide clarity. “Most LBP resolves in SEVERAL weeks’ time, MRIs and X-rays are NOT MUCH of a help, over the counter medications should be used sparingly and narcotics should be AVOIDED COMPLETELY, the two most important things YOU can do for your LBP are to STAY ACTIVE and THINK POSITIVE” Detailed information available within these clickable categories: What’s in a name? Who’s affected? How can exercise help? What else can I do? Signed of a more serious problem Questions to ask my doctor Content reinforces “The Truth About Low Back Pain” video education No attempt made to provide any other information or education to the patient other than what would be normally received were the patient to receive it outside of the study.
**Item 4: WHAT, PROCEDURES** Patients presenting to primary care with a primary complaint of low back pain were contacted by phone and asked to arrive 15 min prior to their scheduled appointment to receive the intervention (watch the video, take the quiz, and review the educational material with the physical therapist)	
**Item 5: WHO PROVIDED** Three licensed physical therapists delivered the education (video, quiz, and educational handout) for all study participants.	
**Item 6: HOW** Participants watched video content with headphones, reviewed materials independently all while in a room with the physical therapist.	
**Item 7: WHERE** Primary Care Clinic Triage Room – adjacent to the front desk and waiting room in the Primary Care clinic of a large military hospital	
**Item 8: WHEN and HOW MUCH** Patient reviewed “The Truth About Low Back Pain” video, completed quiz and complimentary educational handout at their initial appointment only. A link to the video was emailed to the participants on enrollment day to review again at home.	
**Item 9: TAILORING** None	
**Item 10: MODIFICATIONS** None	
**Item 11: HOW WELL, PLANNED** We delivered the intervention in-person on the enrollment day to maximize fidelity. The quiz was given after watching the video to ensure the key messages were highlighted, and before engaging with the patient to answer any additional questions potentially raised and not answered in the video and quiz.	
**Item 12: HOW WELL, ACTUAL** Because the intervention was delivered only once, at baseline, it was delivered as planned	

### Outcomes

The primary outcome was low-value medical utilization (x-rays, MRIs, opioid prescriptions, injections, procedures, etc.) that took place as the initial treatment option within the first 7 days of care. If no treatment was registered beyond this time point, then patients were classified as having no additional care. Medical utilization was determined from manual review of medical records, as well as extraction from the Military Health System Data Repository by procedural (Current Procedural Terminology - CPT) and diagnostic codes (International Classification of Diseases, 9th and 10th revisions - ICD9 and ICD10). We identified all common pharmacological and non-pharmacological interventions utilized for LBP, to include pharmaceutical analgesics (NSAIDs, acetaminophen, ketorolac injections, opioid-based pain relievers to include tramadol), acupuncture, dry-needling, manual therapy and spinal manipulation, therapeutic exercise, as well as referrals to specialty care (physical therapy, sports medicine, orthopaedics, etc.) and diagnostic procedures (e.g., x-rays, MRI, CT-scan). The supplementary online appendix has the list of procedure and diagnosis codes that were utilized. Outcome assessors were blinded to group allocation at all time points. Medical utilization data was fully extracted from the medical records and from the MDR database by an independent data analyst working for the hospital, blinded to the treatment allocation of each participant.

The secondary outcomes included the change in Patient Reported Outcomes Measurement Information System (PROMIS) scores (pain intensity, pain interference, and physical function subscales) at 1 and 6 months and 1-year total LBP-related medical costs. As psychosocial risk factors can drive healthcare utilization for low back pain, we also captured the risk of poor prognosis using the STarT Back Screening Tool (SBST) [30] and the Optimal Screening for Prediction of Referral and Outcomes Yellow Flag (OSPRO-YF) tool [31] at baseline. The SBST tool is a 9-item tool that classified patients into low, medium, and high risk for poor long-term outcome. The OSPRO-YF is a 10-item tool that identifies the presence of 11 “yellow flags” that represent psychosocial risk factors within three distinct domains: negative mood, positive affect and coping, and fear avoidance [31].

#### Patient satisfaction

Patient satisfaction with the care each received for LBP was measured for each participant using a 17-item instrument that has been validated and found capable of distinguishing among three different dimensions of satisfaction (caring, information and treatment effectiveness) among patients with LBP attending primary care [32]. Each item asks about satisfaction on a 5-point Likert scale from 1 = strongly agree to 5 = strongly disagree.

#### Data source

Data were sourced from the Military Health System Data Repository (MDR), which captured data from 260 sources worldwide for any individual covered by the TRICARE insurance program. This includes data from all outpatient and inpatient encounters, in civilian and military hospitals and clinics, pharmacy and radiology data, for military service members, their dependents and family members, and retired service members and their families all around the world. Data is continuously validated for 90 days, with updates from many sources, before variables are converted from ‘raw’ to ‘final’. Data for this study was extracted after 90 days from the last date of interest to ensure maximum validity of data.

### Statistical approach

There were various healthcare utilization outcomes of interest, captured as dichotomous measures of occurrence (YES or NO). We calculated that a sample size of 206 patients would provide the trial with 80% power, at a two-sided alpha level 0.05 and a Cohen’s W of 0.25 for a small effect size, resulting in a critical χ^2^ of 11.07. The sample-size calculation was performed with the use of G*Power software, version 3.1.9.6 [33]. We added 14 additional patients to account for potential loss to follow-up for a total recruitment goal of 220.

For the primary outcome, we calculated frequency of each type of initial treatment with a chi-square analysis and reported odds ratios with 95% confidence intervals for each care option based on the treatment group of initial randomization. If events happened multiple times (e.g., 2+ physical therapy visits, 2 different opioid prescriptions, etc.) the frequency was counted once. For assessment of change in PROMIS subscales from baseline to 6 months between groups, we utilized a linear mixed effects model (LMM), with Bonferroni adjustment for multiple comparisons (baseline, 1 month, 6 months). We chose the LMM because it is flexible and appropriate in accounting for unbalanced and missing data in repeated measures mixed model designs [34]. We report estimated marginal means with 95% confidence intervals. Participants were analyzed according to the group of initial assignment.

#### Sensitivity analysis

Because prior opioid use is one of the strongest predictors of future opioid use, we conducted a sensitivity analysis excluding all patients with any opioid prescription fills in the prior year.

#### Exploratory analysis

We also conducted an exploratory analysis to assess the influence of psychosocial risk factors on the primary outcome (initial healthcare utilization choices). We utilized the same analysis employed for the primary outcome (initial treatment received) but based on high versus medium/low STarT Back risk or presence of any yellow flags in any of the 3 OSPRO-YF domains regardless of initial treatment randomization assignment.

#### Missing data

For our primary outcome, we didn’t expect any missing values because it was based on healthcare utilization data. This is a closed, single-payer system where all care is covered (essentially a government-sponsored socialized medical system). As many as 3% have other health insurance (most often through spouses), but there is no evidence the other insurance is used and not likely if they initially sought care in this system, especially where there is no co-pay for care. If a healthcare event was not present in the MDR, we assumed it did not happen. For the secondary outcomes, we prespecified the use of our statistical model as the primary plan for handling missing data.

## Results

**Fig. 1:**
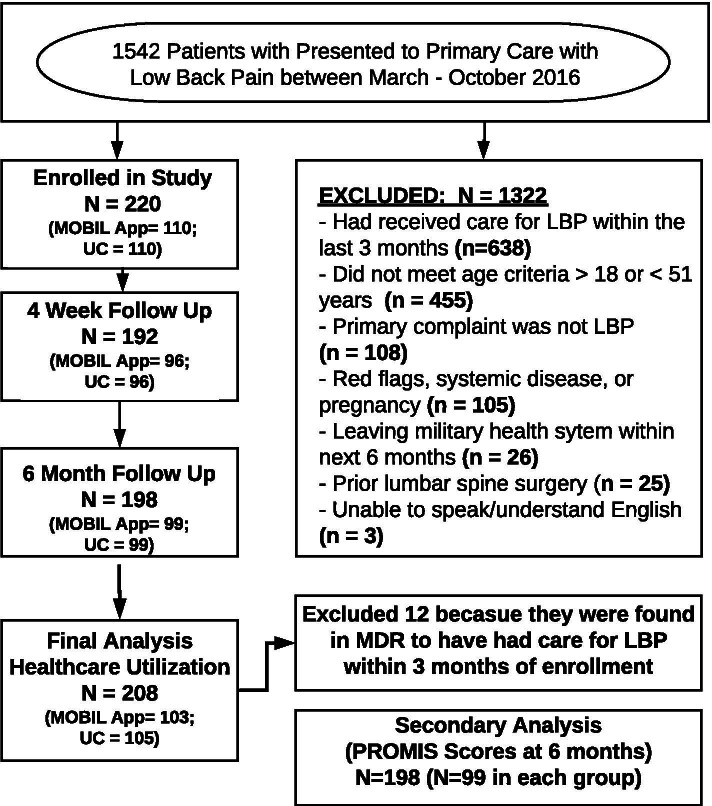
CONSORT Flow Diagram for Trial. Note: LBP = low back pain; MDR = Military Health System Data Repository; PROMIS = Patient-Reported Outcomes Measurement Information System; UC = usual care

**Fig. 2:**
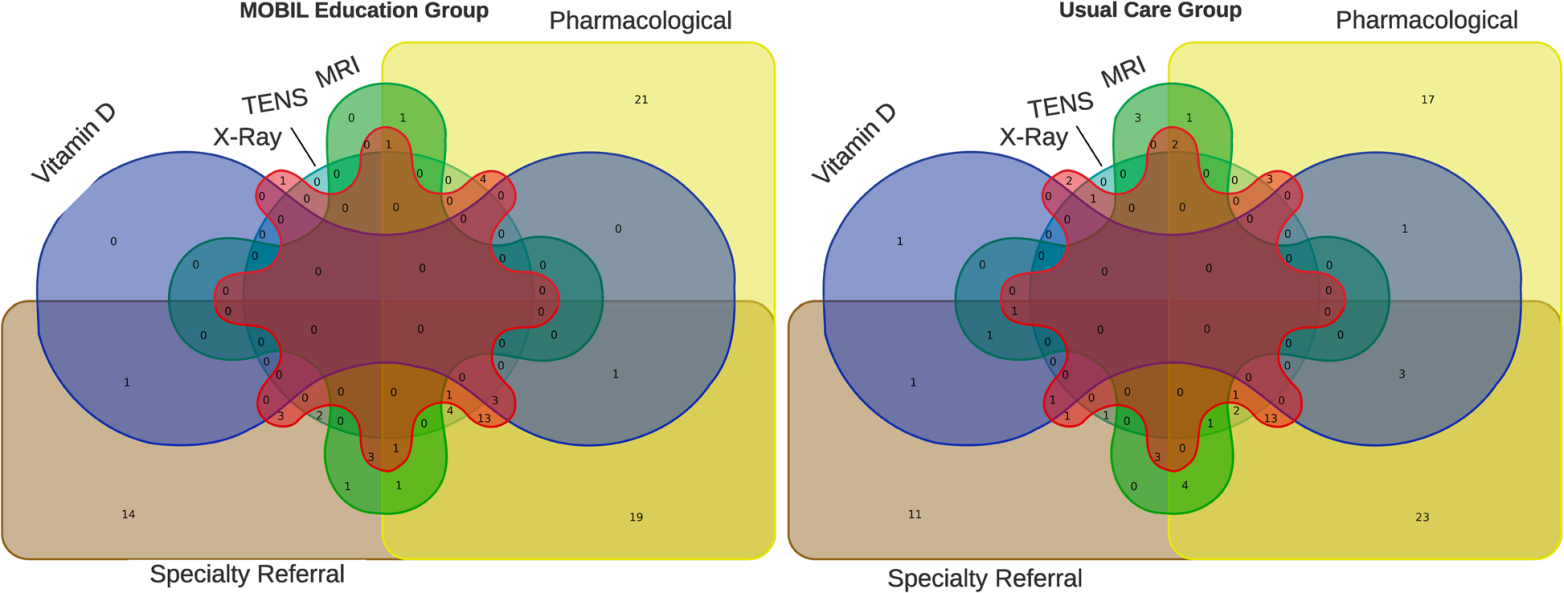
Comparison of all initial treatment choices for LBP based on group assignment. Note: LBP = low back pain; MRI = magnetic resonance imaging; TENS = Transcutaneous Electrical Nerve Stimulation (home-use unit); x-ray = radiograph

**Fig. 3:**
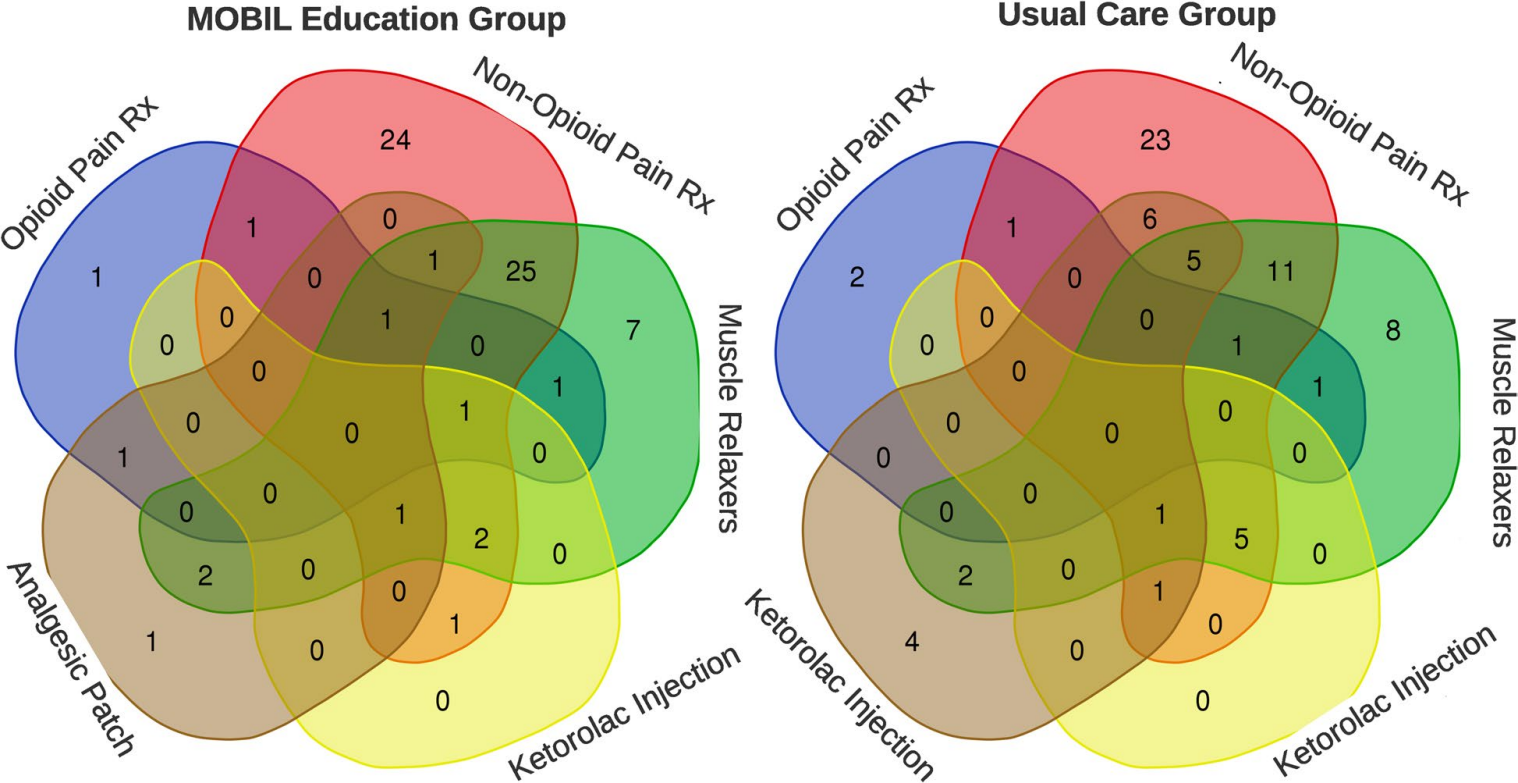
Pharmacological treatments for LBP based on group assignment. Note: LBP = low back pain

There were 220 participants that enrolled between March and October 2016, but 12 were found to have evidence of back-related care in the 3 months prior to enrollment and were excluded from further analyses (Fig. 1). For the 208 participants included (71.2% male; mean age 35.3 years; Table 2), the rates of medication prescription, diagnostic tests, and specialty referrals put into place after the initial consultation were no different between groups (Table 3). The app education session did not influence decisions made by primary care providers compared to usual care alone. Many patients received a combination of non-pharmacological (Fig. 2) and pharmacological treatment (Fig. 3), and only 15 patients received no treatment at all (procedures, pharmacological agents, or referrals). In the sensitivity analyses, the utilization of low-value care was lower for opioid-naïve patients that received the app education, but the differences were not significant.

**Table 2:** Baseline Demographic Variable Comparison Between Groups

		Treatment Group	
	All (*N* = 208)	Usual Care (*N* = 105)	App Education (*N* = 103)
Age – Mean (SD)	35.3 (7.4)	35.4 (7.3)	35.3 (7.6)
Female Sex	60 (28.8)	34 (32.4)	26 (25.2)
Beneficiary Category			
Active Duty/Reserve	167 (80.3)	86 (81.9)	81 (78.6)
Retired Service Member	12 (5.8)	6 (5.7)	6 (5.8)
Dependent	29 (13.9)	13 (12.4)	16 (15.5)
Race			
White	121 (58.2)	60 (57.1)	61 (59.2)
Black or African-American	39 (18.8)	18 (17.1)	21 (20.4)
Asian	10 (4.8)	5 (4.8)	5 (4.9)
Native Hawaiian or Pacific Islander	4 (1.9)	2 (1.9)	2 (1.9)
Other	25 (12.0)	15 (14.3)	10 (9.7)
More than one race	9 (4.3)	5 (4.8)	4 (3.9)
Socioeconomic Status†			
Junior Enlisted	9 (4.3)	4 (3.8)	5 (4.9)
Senior Enlisted	145 (69.7)	73 (69.5)	72 (69.9)
Junior Officer	27 (13.0)	16 (15.2)	11 (10.7)
Senior Officer	27 (13.0)	12 (11.4)	15 (14.6)
Military Service of Sponsor†			
Army	150 (72.1)	73 (69.5)	77 (74.8)
Air Force	32 (15.4)	18 (17.1)	14 (13.6)
Marine Corps	1 (0.5)	0	1 (1.0)
Navy	23 (11.1)	13 (12.4)	10 (9.7)
Coast Guard	2 (1.0)	1 (1.0)	1 (1.0)
Ethnicity			
Hispanic/Latino	59 (28.4)	34 (32.4)	25 (24.3)
Education Level			
High school only	14 (6.7)	4 (3.8)	10 (9.7)
Some college	91 (43.8)	51 (48.6)	40 (38.8)
Graduated from college	56 (26.9)	27 (25.7)	29 (28.2)
Post-Graduate (partial or completion)	47 (22.6)	23 (21.9)	24 (23.3)
Marital Status			
Single	23 (11.1)	13 (12.4)	10 (9.7)
Married or living with significant other	170 (81.7)	82 (78.1)	88 (85.4)
Divorced or Separated	15 (7.2)	10 (9.5)	5 (4.9)
Prior opioid Prescriptions Fills			
No fills in prior year (opioid naïve)	111 (53.4)	49 (46.7)	62 (60.2)
STarT Back Risk Score - Baseline			
Total Score – Mean (SD)	4.23 (2.18)	4.04 (2.13)	4.43 (2.23)
*Risk Stratification Category*			
Low	87 (41.8)	48 (45.7)	39 (37.9)
Medium	83 (39.9)	40 (38.1)	43 (41.7)
High	38 (18.3)	17 (16.2)	21 (20.4)

*Note:* N (%) unless otherwise stated; SD = standard deviation

**Table 3:** Initial Treatments Rendered for Low Back Pain Between Intervention Groups

	Usual Care (N = 105)	App Education Intervention (N = 103)	Odds Ratio^**a**^ (95 CI)
Pharmacological			
Opioid Prescription	5	6	1.24 (0.37 to 4.19)
NSAID Prescription	54	55	1.08 (0.63 to 1.87)
Muscle Relaxer Prescription	34	41	1.38 (0.78 to 2.44)
Ketolodac Injection	7	5	0.71 (0.22 to 2.33)
Analgesic Patch	19	7	**0.33 (0.13 to 0.82)**^**b**^
Vitamin D	9	5	0.54 (0.18 to 1.68)
TENS	6	7	1.20 (0.39 to 3.71)
Specialty Referral	67	67	1.06 (0.60 to 1.86)
*- Physical Therapy*	50	53	
*- Chiropractor*	12	13	
*- Pain Management*	2	5	
*- Orthopaedics*	1	1	
*- Neurosurgery*	3	0	
Diagnostic Imaging			
*- Lumbar X-ray*	28	30	1.13 (0.62 to 2.07)
*- Lumbar MRI*	16	8	0.47 (0.19 to 1.15)
*- X-ray & MRI ordered same day*	6	5	0.84 (0.25 to 2.85)
Received No Care^c^	7	8	1.18 (0.41 to 3.38)
Number of Interventions			
*- 2 or more (*versus *1 or less)*	64	59	0.86 (0.50 to 1.50)
*- 3 or more (*versus *2 or less)*	32	28	0.85 (0.47 to 1.55)
*- 4 or more (*versus *3 or less)*	3	5	1.74 (0.40 to 7.45)

Note: ^a^Reflects the odds of the App Education group in reference to the Usual Care group with bolded values ^b^ being statistically significant; CI = Confidence Interval; NSAID = Non-steroidal Anti-Inflammatory Drug; TENS = Transcutaneous Electrical Nerve Stimulation (prescribed a device to take home); ^c^‘No care’ interpreted as absence of any procedures, prescriptions, or referrals

The changes in PROMIS pain and physical function were also no different between groups at 6 months (physical function mean difference = 0.35, 95CI -2.46, 1.76; pain interference mean difference = 1.05, 95CI -1.27, 3.37; pain intensity mean difference = 0.003, 95CI -0.58, 0.59).

In the exploratory analysis of initial treatment options based on the presence of various psychosocial and prognostic risk factors, most treatment options were given in higher proportion to patients with yellow flags or prognostic risk factors of pain-associated distress; however, the differences were not statistically significant (Table 4). Opioid prescriptions and pharmacological treatments in general (driven primarily by NSAID prescriptions) were significantly higher in patients that were high-risk on the STarT Back screening tool and had fear avoidance beliefs, respectively (Table 4). This study was not powered to assess differences based on these domains, and no definitive inferences can be made. No harm or unintended effect of treatment was reported by any participant in this study.

**Table 4:** Initial treatments rendered – grouped by high prognostic risk or yellow flag factor presence

	Prognostic Risk (STarT Back Screening Tool)			Yellow Flag Domains from OSPRO-YF								
				Negative Mood			Positive Affect and Coping			Fear Avoidance		
	Low/Med (***N*** = 170)	High (***N*** = 38)	OR (95 CI)	YES (***N*** = 111)	NO (***N*** = 97)	OR (95 CI)	YES (***N*** = 153)	NO (***N*** = 55)	OR (95 CI)	YES (***N*** = 162)	NO (***N*** = 46)	OR (95 CI)
Any pharmacological treatment	113	28	1.41 (0.64 to 3.11)	78	63	1.28 (0.71 to 2.28)	109	32	1.78 (0.94 to 3.38)	116	25	**2.12** **(1.08 to 4.15)**
- *Opioid Rx*	6	5	**4.14**^**a**^ **(1.19 to 14.37)**	9	2	4.19 (0.88 to 19.89)	11	0	–	11	0	–
- *NSAID Rx*	89	20	1.01 (0.50 to 2.05)	58	57	0.77 (0.44 to 1.33)	84	25	1.46 (0.79 to 2.71)	91	18	**2.0**^**a**^ **(1.02 to 3.89)**
- *Muscle Relaxers*	61	14	1.04 (0.50 to 2.16)	44	31	1.40 (0.79 to 2.48)	56	19	1.19 (0.62 to 2.26)	59	16	1.07 (0.54 to 2.13)
- *Toradol Injection*	10	2	0.89 (0.19 to 4.23)	7	5	1.24 (0.38 to 4.04)	10	2	1.85 (0.39 to 8.74)	11	1	3.28 (0.41 to 26.08)
- *Analgesic Patch*	20	6	1.41 (0.52 to 3.78)	11	15	0.60 (0.26 to 1.38)	19	7	0.97 (0.38 to 2.46)	21	5	1.22 (0.43 to 3.44)
- *Vitamin D*	11	3	1.24 (0.33 to 4.68)	11	3	3.45 (0.93 to 12.74)	9	5	0.63 (0.20 to 1.95)	11	3	1.04 (0.28 to 3.91)
TENS	11	2	0.80 (0.17 to 3.78)	4	9	0.37 (0.11 to 1.23)	8	5	0.55 (0.17 to 1.76)	11	2	1.04 (0.28 to 3.91)
Specialty Referral	109	25	1.08 (0.51 to 2.26)	68	66	0.74 (0.42 to 1.32)	97	37	0.84 (0.43 to 1.62)	106	28	1.22 (0.62 to 2.39)
*- Physical Therapy*	87	16		50	53		77	26		84	19	
*- Chiropractor*	20	5		13	12		14	11		18	7	
*- Pain Management*	3	4		4	3		6	1		5	2	
*- Orthopaedics*	1	1		2	0		2	0		1	1	
*- Neurosurgery*	1	2		3	0		3	0		3	0	
Diagnostic Imaging												
*- Lumbar X-ray*	50	8	0.64 (0.27 to 1.49)	31	27	0.63 (0.35 to 1.13)	46	12	1.54 (0.74 to 3.19)	49	9	1.78 (0.80 to 3.98)
*- Lumbar MRI*	17	7	2.03 (0.78 to 5.31)	16	8	1.87 (0.76 to 4.59)	16	8	0.69 (0.28 to 1.71)	21	3	2.13 (0.61 to 7.50)

Note: The OR reflects the odds of the high risk group or presence of yellow flag in reference to the low/medium risk group or absence of yellow flag, with bolded values ^a^ being statistically significant; OSPRO-YF = Optimal Screening for Prediction or Referral and Outcome Yellow Flag; OR = Odds Ratio; NSAID = Non-steroidal Anti-inflammatory Drugs

Patient satisfaction with the care they received was not different between groups (Table 5). The mean overall score and standard deviation for all 17 questions was 54.0 (6.0) for the usual care group and 54.6 (3.7) for the education group. All three of the subscale scores were also similar between both groups.

**Table 5:** Patient Satisfaction with Care Received

Patient Satisfaction Scale scores – mean (standard deviation)	Usual Care (N = 105)	App Education Intervention (N = 103)
Overall Score (range of 17 to 85 points)	54.0 (6.0)	54.6 (3.7)
**Subscale Scores**		
Satisfaction with Information from Provider (range of 3 to 30 points)	7.0 (2.5)	7.4 (2.8)
Satisfaction with Caring of Provider (range of 4 to 40 points)	6.9 (2.9)	6.9 (3.1)
Satisfaction with Effectiveness of Provider (range of 3 to 30 points)	7.2 (2.1)	7.4 (2.4)

Note: Answers for each question range from 1 = strongly agree to 5 = strongly disagree

## Discussion

Equipping patients with proper education about best care practice and expectations for low back pain within the context of a biopsychosocial framework had no influence on treatment decisions made by primary care providers compared to patients not getting the education. Any actual or perceived pressure from patients to make care decisions that contradict guideline-adherent care was unchanged with this educational approach.

However, psychosocial beliefs associated with greater risk for poor prognosis were common in this cohort of patients seeking initial care for LBP in primary care settings. Over half of the patients had at least 1 yellow flag on the OSPRO-YF and approximately 1 in 5 were considered high-risk on the SBST. Regardless of beliefs, there was large variability in the care delivered, much of which would not be considered high-value or concordant with current clinical practice guidelines [35, 36]. Initial treatments were not significantly different for patients that received the biopsychosocial risk-focused app interaction prior to their visit with the primary care clinician compared to those that did not receive the app. Education on self-management is key as an early intervention, with a strong focus on addressing maladaptive beliefs, while routine imaging and opioid-based pain medications are not recommended as initial treatment strategies [11, 35]. In this study, initial care for a new episode of back pain consisted of 134 (64.4%) receiving a specialty care referral, 58 (27.9%) having an x-ray ordered, and 24 (11.5%) having an MRI ordered (Fig. 2). For pharmacological interventions, 109 (52.4%) received NSAIDs, 75 (36.1%) received muscle relaxers, and 11 (5.2%) received opioids (Fig. 3). Patients also received analgesic patches, vitamin D supplementation, and transcutaneous electrical stimulation (TENS) units for home use (Figs. 2 and 3), all treatments with unknown or little efficacy.

Despite a high proportion of individuals with maladaptive psychosocial beliefs at baseline, patient-focused education did not result in any changes in treatment decisions made by primary care providers. A prior study in military soldiers found that a brief educational session focused on addressing psychosocial beliefs, and delivered in group format, resulted in decreased healthcare seeking for low back pain in the following year [37]. However, all were healthy without LBP at the time of the education, and therefore the decisions related to whether they should seek care in the first place were different than those about what intervention should be received after the decision has been made to seek care. They also would have had additional time to digest the information before seeing a medical provider. Perhaps more time to mentally process the information (instead of immediately before their consultation) would lead to greater advocacy for high-value care. While education has been targeted at clinicians with mixed results in the past [25], the clinicians in our trial were blinded to the treatment arm and entire educational component for the duration of the study. It is possible that some providers adapt to routines for managing specific conditions, where passive patient personalities are less likely to challenge any decisions made by clinicians. Patients generally want to be actively involved in management decisions for LBP [38], but without the clinician being aware of the education the patient received, they would be limited in their ability to engage and continue the conversations that were started. One study found that focusing psychosocial education and training on medical students and general practitioner trainees can likely influence treatment decisions [39]. For our study, revealing the group allocation would have biased providers and likely confounded the impact of the educational app session. Bringing the clinician into the education process earlier would likely have great merit and should be considered in future studies. Receiving the education from their general practitioner, with whom they may already have a stronger rapport, could also influence its impact. It could also be that the clinicians themselves are providing conflicting or predominately biomedically-focused information, and the patient must now balance what they learned in the video education app versus what they are hearing from the provider.

Similar educational programs focused on targeting the psychosocial component behind treatment decisions have had varying levels of success. Mass media campaigns have been shown to positively influence both patient and provider beliefs about LBP [24], but it is unclear if the change in beliefs results in changes in clinical practice. In addition, while decisions appear to be improved when dealing with vignettes [39], real-life scenarios may have different outcomes. In emergency medicine settings, Choosing Wisely campaigns were able to significantly improve knowledge and awareness of guideline recommendations for LBP imaging, however it did not translate into any actual reduction in imaging rates [25]. In 6 large primary care clinics where clinicians committed ahead of time to follow guidelines, there were only minor changes (1.2 to 1.9%) in low-value care decisions that were not sustained in the long-term [40].

The psychosocial profiles of patients and their relation to higher utilization of treatment needs further investigation. Targeting only patients at high risk for poor prognosis (psychosocial risk factors present) may be necessary to truly understand the value of these patient or provider-focused education interventions. The effect may be diluted with the inclusion of all patients (low and high risk). This study was not powered to fully detect those differences, which could explain why medical utilization was higher in patients presenting with psychosocial risk factors, but the differences were not significant. Other studies have shown that high rates of fear avoidance [41] beliefs and pain catastrophizing result in higher healthcare utilization [42, 43]. Identifying high-risk individuals to prioritize for this type of education could be beneficial. At the same time, the minimal cost and effort to deliver this care to everyone is likely sustainable and could help create a better culture of information within specific clinical settings.

### Strengths and limitations

The strengths of this study include the moderate sample size and long-term follow-up, as well as the closed single-payer system which allows essentially complete capture of all healthcare utilization events. This is also a potential weakness, as a single-payer government setting, these findings from the Military Health System may not be representative of results seen in other settings. It is possible that some patients revealed or shared with the primary care provider the content and nature of the video app, which may have introduced bias (the clinician would now know about the education patients are receiving and ask future patients what they received). There was a larger proportion of patients in the app education group that had prior opioid use (60.2% versus 46.7%) which may have influenced the opioid outcomes as that is a strong predictor of future opioid use. Secondary analyses of subgroups (high risk and presence of yellow risk factors) were all performed with an inappropriately powered sample size, which limits any conclusions.

## Conclusion

Factors that influence medical decisions and guideline-concordant care are complex. Despite many patients with LBP having maladaptive psychosocial beliefs at baseline, an attempt to address those beliefs at the point of care with the patient through an innovative software app interaction did not change initial treatment decisions made by the clinician. This particular patient education approach did not appear to influence healthcare decisions. Future studies should consider focusing exclusively on high-risk populations, on a simultaneous focus of both the patient and clinician, and on the impact of including the medical provider in the educational experience from the beginning.

## Data Availability

Reasonable requests for data will be considered after meeting proper data sharing agreement requirements from the US Defense Health Agency (DHA) (Data Sharing Agreement Applications [DSAA] and templates can be found at health.mil). After DSA approval by the DHA, please contact the corresponding author for data.
